# Dual Inhibition of B7-H3 and EGFR Overcomes Acquired Chemoresistance in Colon Adenocarcinoma

**DOI:** 10.7150/jca.91089

**Published:** 2024-02-04

**Authors:** Liang-Chi Chen, Pei-Chen Yang, Chia-Yi Chen, Shu-Fen Chiang, Tsung-Wei Chen, William Tzu-Liang Chen, Tao-Wei Ke, Ji-An Liang, An‑Cheng Shiau, K. S. Clifford Chao, Kevin Chih-Yang Huang

**Affiliations:** 1 Department of Pathology, China Medical University Hospital, China Medical University, Taichung 40402, Taiwan.; 2 Proton Therapy and Science Center, China Medical University Hospital, China Medical University, Taichung 40402, Taiwan.; 3 Lab of Precision Medicine, Feng-Yuan Hospital, Ministry of Health and Welfare, Taichung 42055, Taiwan.; 4 Department of Pathology, Asia University Hospital, Asia University, Taichung 41354, Taiwan.; 5 Department of Colorectal Surgery, China Medical University HsinChu Hospital, China Medical University, HsinChu 302, Taiwan.; 6 Department of Colorectal Surgery, China Medical University Hospital, China Medical University, Taichung 40402, Taiwan.; 7 Department of Surgery, School of Medicine, China Medical University, Taichung 40402, Taiwan.; 8 School of Chinese Medicine, China Medical University, Taichung 40402, Taiwan.; 9 Department of Radiation Oncology, China Medical University Hospital, China Medical University, Taichung, Taiwan.; 10 Department of Radiotherapy, School of Medicine, China Medical University, Taichung 40402, Taiwan.; 11 Department of Biomedical Imaging and Radiological Science, China Medical University, Taichung 40402, Taiwan.; 12 Translation Research Core, China Medical University Hospital, China Medical University, Taichung 40402, Taiwan.; 13 Cancer Biology and Precision Therapeutics Center, China Medical University, Taichung 40402, Taiwan.

**Keywords:** Colorectal cancer, B7-H3, Intratumoral infiltrating lymphocytes, PD-L1

## Abstract

Despite advances in therapeutic strategies for colorectal cancer (CRC), CRC has a high disease incidence with significant morbidity and mortality worldwide. Notably, immunotherapy has shown limited efficacy in treating metastatic CRC, underscoring the need for alternative immunotherapeutic targets for the management of metastatic colorectal cancer (mCRC). In the present study, we evaluated the levels of the immune checkpoint proteins PD-L1, PD-L2 and B7-H3 in a large cohort retrospective study. We found that tumor B7-H3 (52.7%) was highly expressed in primary tumors compared to that in PD-L1 (33.6%) or PD-L2 (34.0%). Elevated B7-H3 expression was associated with advanced stage and the risk of distant metastasis and correlated with poor disease-free survival (DFS), suggesting that tumor B7-H3 was an independent prognostic factor associated with worse DFS in colon adenocarcinoma patients (COAD), especially high-risk COAD patients who received adjuvant chemotherapy. Furthermore, we found that B7-H3 significantly promoted cell proliferation and tumor growth in CRC. B7-H3 may stabilize EGFR to activate its downstream pathway for cancer cell proliferation and resistance to oxaliplatin (OXP). Dual targeting of B7-H3 and EGFR markedly rescued the susceptibility to chemotherapy in colorectal cancer cells *in vitro* and *in vivo*. Overall, these results showed that B7-H3 exhibited a high prevalence in COAD patients and was significantly associated with worse prognosis in COAD patients. Dual targeting of B7-H3 and EGFR signaling might be a potential therapeutic strategy for high-risk COAD patients.

## Introduction

Colorectal cancer (CRC) ranks third and second in morbidity and mortality worldwide, respectively 1. Despite the prevalence of cancer screening in CRC, over 25% of CRC patients are diagnosed at an advanced stage with metastases, and ~50% of CRC patients eventually develop metastases after surgery during long-term follow-up 2. Although multimodal therapies, including surgery, chemotherapy, radiotherapy and targeted therapy, have improved survival in advanced CRC patients, the 5-year survival of metastatic CRC (mCRC) patients remains low, at ~12-14% 3, 4. Consequently, there is an urgent need for novel therapeutic strategies to treat metastatic CRC patients, including the potential use of immune checkpoint inhibitors (ICIs).

One crucial member of the B7-CD28 immune checkpoint family is B7-H3 (B7 homology 3, CD276). B7-H3 is a type I transmembrane protein that plays a pivotal role in T-cell-mediated immune responses 5-7. Additionally, B7-H3 is highly expressed in various malignancies, including breast, pancreatic, hepatocellular, and lung cancers and CRC 8-13. High B7-H3 expression has been associated with tumor progression and poor overall survival in different types of malignancies 14-16, including CRC 13, 17, 18. In addition to its immunologic function, B7-H3 is involved in various cellular processes, such as cell growth, migration, invasion, epithelial-to-mesenchymal transition (EMT), and cancer stemness 19. Evidence suggests that B7-H3 may contribute to tumor initiation and the acquisition of tumor aggressiveness within specific cellular microenvironments. Notably, B7-H3 can promote EMT by modulating the expression of key markers such as E-cadherin, β-catenin, N-cadherin and vimentin 20. Furthermore, B7-H3 affects the sensitivity of various anticancer drugs and targeted therapies in multiple cancer types, including CRC 21-24. B7-H3 can upregulate the expression of DNA repair-associated proteins, such as BRCA1/BRCA2-containing complex subunit 3 (BRCC3) or X-ray repair cross-complementing Group 1 (XRCC1), to counteract DNA damage caused by agents such as 5-fluorouracil (5-FU) or oxaliplatin (OXP) 22, 25. Anti-B7-H3-drug conjugates have been shown to target both tumor cells and tumor vasculature in human CRC xenografts 14. Although these studies have elucidated the various roles of B7-H3 in CRC, there remains a need for a comprehensive understanding of the underlying mechanisms by which B7-H3 mediates chemoresistance in human CRC.

Recent studies showed that targeting B7-H3 by chimeric antigen receptor T cells exhibited superior and safe antitumor effects 26, 27, indicating that B7-H3 is crucial for modulating the antitumor immune response and that targeting B7-H3 is a potential immunotherapeutic strategy for cancer treatment. In our study, we aimed to comprehensively assess the prognostic significance of tumor B7-H3 expression in primary tumors and their respective recurrent metastatic sites. Our findings revealed that high levels of tumor B7-H3 are associated with poorer survival outcomes in patients with colon adenocarcinoma (COAD), especially in high-risk COAD patients who have received postoperative chemotherapy. Furthermore, we uncovered that tumor-intrinsic B7-H3 expression enhanced cell growth and tumor growth via the EGFR signaling pathway *in vitro* and *in vivo*. Tumor-intrinsic B7-H3 also promotes resistance to OXP via EGFR signaling. Dual inhibition of B7-H3 and EGFR significantly increased the susceptibility of B7-H3-expressing CRC to OXP *in vitro* and *in vivo*. In summary, our results suggest that B7-H3 can serve as an independent prognostic factor for COAD patients who receive adjuvant chemotherapy, and dual targeting of the B7-H3 and EGFR signaling pathways may be a promising strategy for treating B7-H3-expressing COAD patients.

## Materials and Methods

### Patients, cohorts and tissue microarray construction

Four hundred twenty-nine stage I-IV colon adenocarcinoma (COAD) patients who underwent initial surgical resection at China Medical University Hospital (CMUH) between 2006 and 2014 were randomly selected. COAD patients who received preoperative chemotherapy, radiotherapy and targeted therapy were excluded from this cohort. Clinical information, including age, sex, primary tumor location, tumor histology, tumor grade, *RAS* mutation status, recurrence status, and survival, was obtained from the cancer registry database in CMUH, which was approved by the Institutional Review Board (IRB). Undefined clinicopathological data were labeled as unknown. The *KRAS* mutation status was mainly carried out in stage III-IV COAD patients (n=154), and the expression of EGFR was randomly evaluated in this cohort (n=144). Tumor stage was redefined according to the 7^th^ AJCC TNM staging system.

A total of 225 patients were men (52.4%), and 204 (47.6%) were women, with a mean age of 64.1±13.9 years. A total of 254 COAD patients received postoperative chemotherapy after surgery, including adjuvant chemotherapy for high-risk stage II-III COAD patients (n=181) and palliative chemotherapy for stage IV metastatic COAD patients (n=73). After treatment, patients were regularly followed up with computed tomography (CT) scanning to detect recurrence according to the routine daily practice of CMUH. The primary and recurrent tumor samples of COAD were used for TMA construction and immunohistochemical (IHC) analyses.

For TMA construction, the standard protocol was followed according to our previous studies 28, 29. Briefly, hematoxylin/eosin (HE)-stained tissue slides were evaluated by pathologists, and the corresponding formalin-fixed, paraffin-embedded tissues were used for TMA construction. Correspondent adjacent normal and tumor tissues were punched from the donor block and transferred into one recipient paraffin block. The primary endpoints of this study were overall survival (OS) and disease-free survival (DFS), which were defined as the time from the surgical day to the event day, including tumor relapse and death.

### Immunohistochemical analysis

The TMA recipient blocks were cut into 3-μm sections for IHC staining with a standard protocol 28, 29. TMA slides were deparaffinized and rehydrated through a series of alcohols before being exposed to the *Antigen Unmasking Solutions* (H3300, Vector Laboratories, Burlingame, CA). Endogenous peroxidase was naturalized with 0.3% hydrogen peroxide for 15 min at room temperature before antibody incubation. Following incubation with the primary rabbit monoclonal B7-H3 antibody (#D9M2L, 1:100, Cell Signaling Tech.), PD-L1 antibody (ab205921, 1:100, Abcam, Cambridge, UK) 29, PD-L2 (ab200377, 1:100, Abcam, Cambridge, UK) 28 and EGFR (IR211-769, iReal Biotechnology) at room temperature for 2 hours. The sections were stained according to the manufacturer's manual (VECTASTAIN Elite ABC Kit, Vector Laboratories), incubated with the substrate DAB chromogen (Vector Laboratories), and then counterstained with hematoxylin 30, 31. Membranous tumor B7-H3 and stromal B7-H3 expression was evaluated based on immune positivity of cells on the membrane of tumor cells for histo-score (H-score) according to the intensity by semiquantitative scale (0 for absent; 1 for weak; 2 for moderate; and 3 for strong membrane staining) and the percentage of membranous tumor B7-H3 or stromal B7-H3 cells. The range of the H-score was from 0 to 300. The definition for high or low tumor B7-H3 was based on the mean expression of B7-H3 expression. For tissue evaluation, the tumor tissue area < 20% TMA spot was excluded and defined as missing data.

### The Cancer Genome Atlas (TCGA)

Stage I-IV CRC patients were included, and their B7-H3 (*CD276*) mRNA expression data were retrieved from the Human Protein Atlas (HPA, www.proteinatlas.org/pathology) 32-34, which is derived from RNA sequencing data and clinical information from TCGA. The *CD276* mRNA level gene had the best log-rank *p* value based on Kaplan‒Meier (KM) analysis with average RNA expression levels as the cutoff (Best expression cutoff =18.92) by the algorithm on the HPA website 34. RNA-seq data for 597 CRC samples derived from primary tumors were obtained from the TCGA-COAD dataset 32, 33. Patients were split into low and high *CD276* according to the cutoff expression of total patients.

### Immunoblotting analysis

Total lysates (30 μg) were separated via 6%-12% SDS‒PAGE, transferred onto PVDF membranes (Millipore, MA, USA) 30, 35, blocked with 5% nonfat milk, incubated with specific antibodies (in 1% nonfat milk) overnight at 4 °C, and probed with HRP-conjugated secondary antibodies. The blot membrane was then incubated with Immobilon Western Chemiluminescent HRP Substrate (Millipore, MA, USA), analyzed by an ImageQuant™ LAS 4000 biomolecular imager (GE Healthcare, Amersham, UK), processed with Adobe Photoshop, and quantified by using ImageJ software (NIH, MD, USA). Each blot was stripped with immunoblotting stripping buffer (BioLion Tech., Taipei, Taiwan) before incubation with the other antibodies.

### Gefitinib and oxaliplatin treatment

BALB/c nude mice (female, 4 weeks old) were maintained according to the institutional guidelines approved by the China Medical University Institutional Animal Care and Use Committee [Protocol No. CMUIACUC-2018-167]. Briefly, CT26-Vec. and CT26-B7-H3 cells (5 × 10^5^ cells/mouse) were suspended in 100 μL of 50% Matrigel and inoculated subcutaneously into the right leg of each mouse. After 7 days, the mice were administered OXP (5 mg/kg) and gefitinib (50 mg/kg) on the indicated days. The longest and shortest diameters (L and W, respectively) of the tumors were measured using Vernier calipers (Sata, Shanghai, China) every 3 days, and tumor volume (V) was calculated by the formula: V = (L × W^2^)/2. The mice were sacrificed at the end of the experiments, and the tumor tissues were collected for lysis, subjected to immunoblotting analysis and stained by immunohistochemistry 36, 37.

### Statistical analysis

The statistical analyses were performed with SPSS software version 22 (SPSS, Chicago, IL, USA) with a *p* value <0.05 as the statistically significant cutoff. Patient survival was estimated by the Kaplan‒Meier log-rank test. The correlation analysis between B7-H3 expression (H-score) and clinicopathological parameters was analyzed by the chi-squared test or Fisher's exact test (two-tailed). Univariate and multivariate Cox regression models were constructed with influencing factors, including pT stage, pN stage, lymphovascular invasion, perineural invasion, tumor B7-H3 and tumor PD-L1, with an entry criterion of *p* < 0.05.

## Results

### The association between clinicopathological characteristics and tumor B7-H3 expression in COAD patients

**Figure 1:**
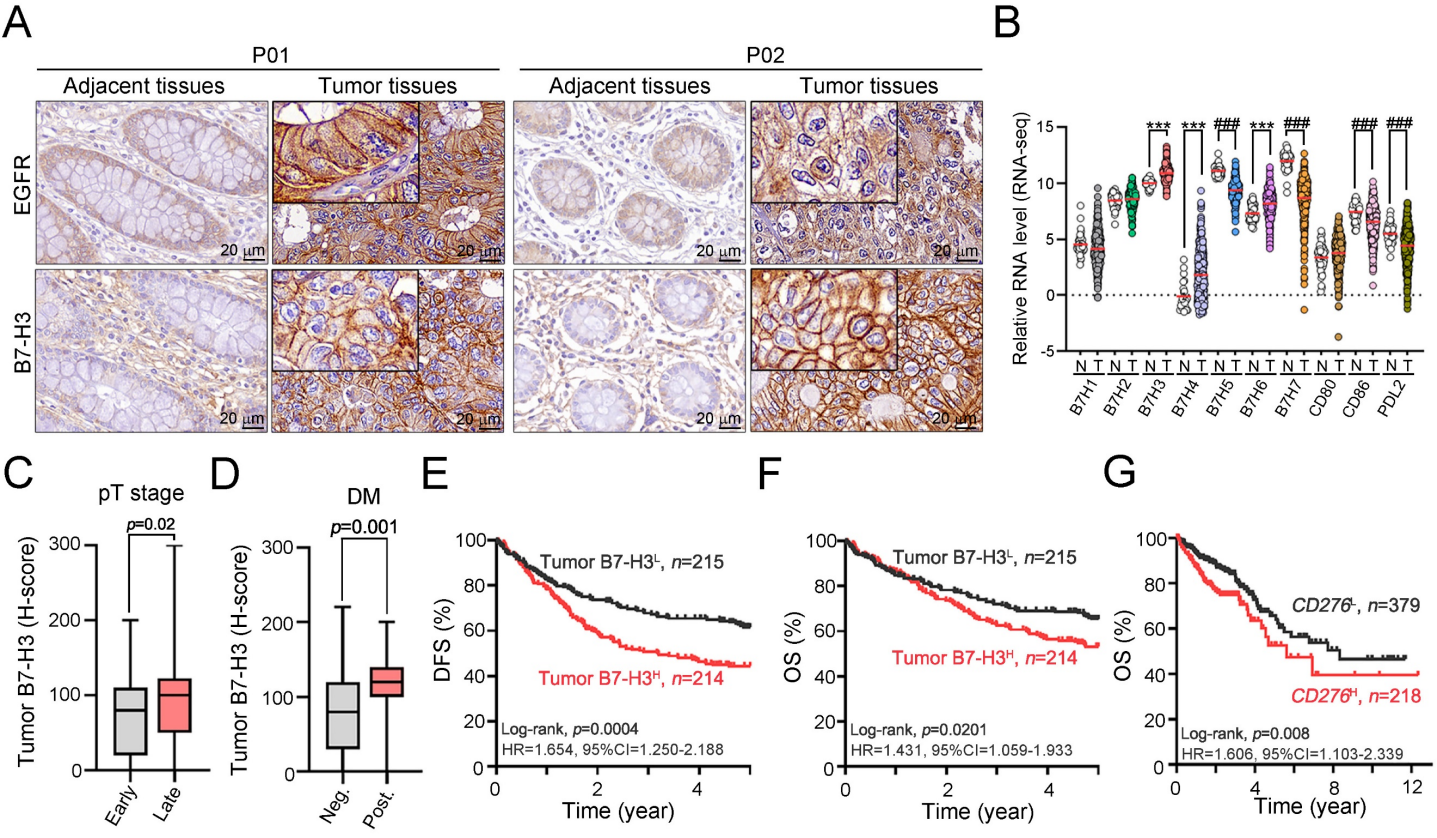
** The expression of tumor B7-H3 was significantly associated with the tumor aggressiveness and survival outcome.** A. B7-H3 proteins were mainly expressed on the membrane of cancer cells and partially expressed in the stromal cells. B. B7-H3 expression was higher than any other B7-CD28 family members. ***p<0.001. C. The H-score of tumor B7-H3 was statistically associated with tumor size (*n*=429, *p*=0.02). Early: pT1-2 and Late: pT3-4. D. Tumor B7-H3 expression was associated with risk of distant metastasis (*n*=429, *p*=0.011). E. Low tumor B7-H3 expression was significantly associated with favorable 5-year disease-free survival (DFS, *n*=429, Log-rank *p*=0.0004). F. Stratifying the level of tumor B7-H3 by mean expression (H-score), low tumor B7-H3 expression was significantly associated with favorable 5-year overall survival (OS, *n*=429, Log-rank *p*=0.02). G. Low *CD276* mRNA was significantly associated with favorable survival outcome (OS, *n*=597, Log-rank *p*=0.008).

We first examined the expression status of B7-H3 protein in 429 cases of colon adenocarcinoma patients who received surgery at China Medical University Hospital between 2006 and 2014 in a retrospective cohort study. We scored the level of membranous B7-H3 on both tumor and stromal cells by semiquantitative scale (0 for absent; 1 for weak; 2 for moderate; and 3 for strong membrane staining, Fig. S1). We used the sum of the density and frequency of membranous B7-H3 expression for further analyses. Representative staining of tumor B7-H3 in COAD tissue is shown in Fig. 1A. As shown in Fig. 1A, B7-H3 was detectable on cancer cells and stromal cells. The clinicopathological and molecular characteristics according to the status of tumor B7-H3 expression are summarized in Table S1. We found that B7-H3 was highly expressed in 49.9% (214/429) of cancer cells and 52.7% (226/429) of stromal cells. These results showed that B7-H3 exhibited higher expression within the TME in COAD patients. Analysis of the mRNA levels of B7 family members in CRC from the TCGA database also showed that B7-H3 exhibited the highest expression among B7 family members (Fig. 1B), indicating that B7-H3 may be a potential therapeutic target. High tumor B7-H3 expression was significantly associated with pT stage (*p* = 0.02, Fig. 1C and Table S1), lymph node metastasis (*p*=0.027, Table S1), lymphovascular invasion (LVI, *p* = 0.035, Table S1), distant metastasis (*p*=0.001, Fig. 1D) and EGFR expression (*p*=0.019, Table S1).

### Elevated tumor B7-H3 expression is significantly associated with unfavorable 5-year DFS and 5-year OS in colon adenocarcinoma

We then examined the prognostic value of tumor B7-H3 expression in colon adenocarcinoma by Kaplan-Meier (KM) analysis. The KM survival analysis showed that low tumor B7-H3 expression was markedly associated with favorable 5-year DFS (62.8% vs. 45.1%, *p*<0.001, Fig. 1E) and 5-year OS (66.5% vs. 54.2%, *p*=0.02, Fig. 1F and Table S2) in stage I-IV patients. By analyzing the *CD276* mRNA expression and clinical outcome results from The Cancer Genome Atlas (TCGA) (stage I-IV, n = 597, Fig. 1G) 33, we found that CRC patients with low *CD276* mRNA had favorable survival outcomes (log-rank *p*=0.008, HR=1.606, 95% CI=1.103-2.339), which was consistent with our results. The cutoff of *CD276* mRNA level for KM analysis was automatically calculated based on the algorithm on the HPA website (www.proteinatlas.org/pathology, best expression cutoff =18.92) 33. The expression of tumor PD-L1 was positively correlated with the MMR status (*p*=0.001, Table S1). However, there was no significant association between B7-H3 and MMR status (*p*=0.466, Table S1).

**Figure 2:**
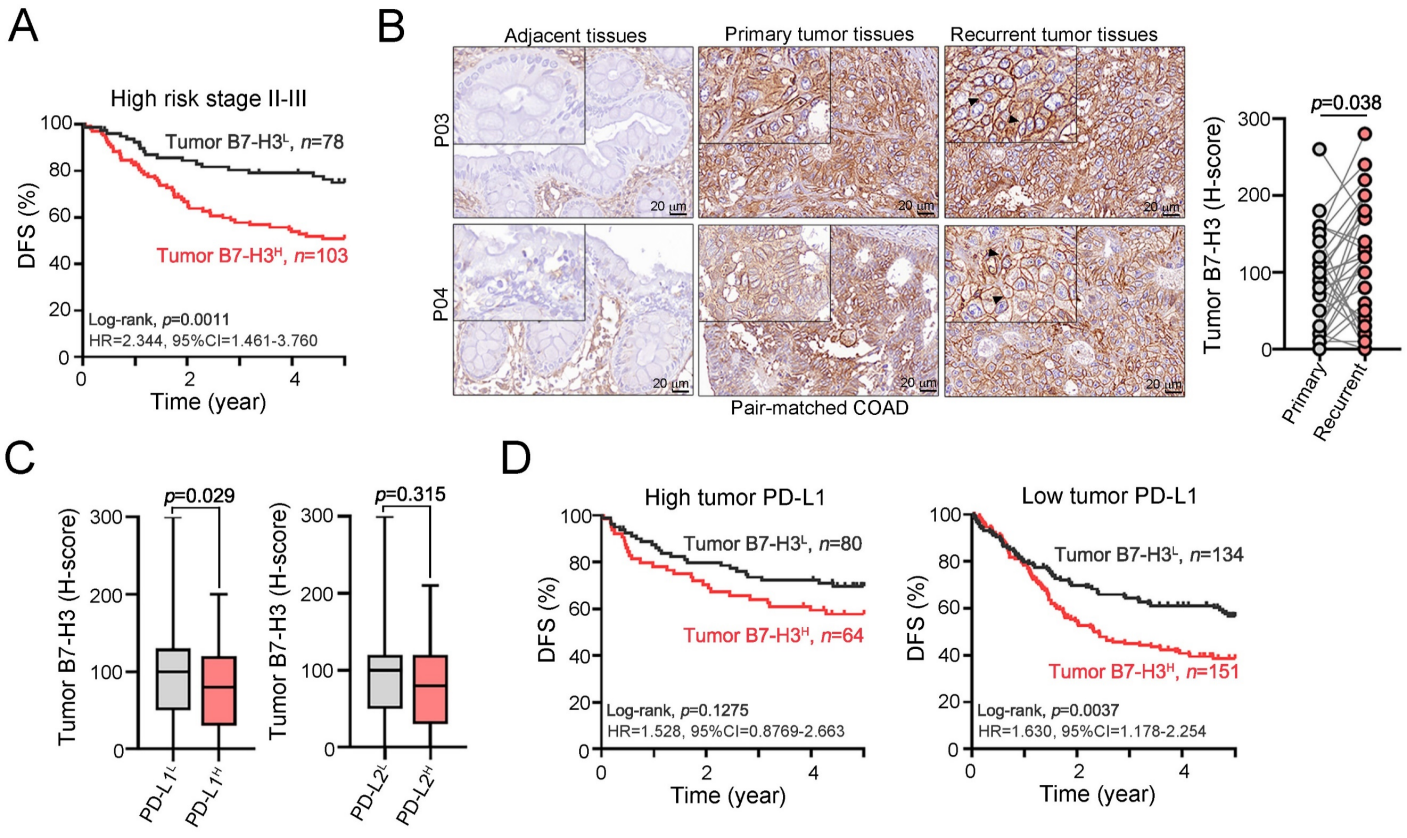
** Recurrent liver metastasis after postoperative chemotherapy showed higher tumor B7-H3 expression.** A. High B7-H3 expression was remarkably associated with poor survival outcome in high-risk stage II-III COAD patients (*n*=181, *p*=0.0011). B. After post-operative adjuvant chemotherapy, recurrent tumor exhibited high level of B7-H3 compared to primary tumors (*n*=38, p=0.038). C. The expression of tumor B7-H3 was negatively associated with tumor PD-L1 expression. D. Stratifying the level of tumor PD-L1, high tumor B7-H3 expression was significantly associated with unfavorable 5-year overall survival in low tumor PD-L1 expressing COAD patients.

Furthermore, when classifying COAD patients into high-risk stage II-III patients based on the postoperative adjuvant chemotherapy regimen, we found that high-risk stage II-III COAD patients with elevated tumor B7-H3 tended to have poorer 5-year DFS than those with low tumor B7-H3 (Fig. 2A and Table S2). We found that elevated tumor B7-H3 expression was observed in metastatic tumors after adjuvant chemotherapy compared to that in primary tumors in pair-matched tumor tissues, suggesting that B7-H3 may limit the therapeutic efficacy of adjuvant chemotherapy (Fig. 2B). Additionally, our previous studies found that tumor PD-L1 and PD-L2 expression was associated with favorable outcomes in COAD patients due to compensatory stimulation of IFNγ^+^CD8^+^ T cells within the tumor microenvironment 28, 29. Therefore, we evaluated the relationship between B7-H3 and PD-L1/PD-L2 in COAD patients. We found a negative association between B7-H3 and PD-L1 (Fig. 2C). The mean tumor expression of B7-H3 was higher in patients with low tumor PD-L1 expression (Fig. 2C). There was no association between B7-H3 and PD-L2 (Fig. 2C). By stratifying COAD patients by the level of PD-L1, we found that high tumor B7-H3 expression was markedly associated with unfavorable 5-year DFS in low PD-L1-expressing COAD patients (Fig. 2D). These results indicated that tumor B7-H3 is significantly associated with the risk of tumor relapse and chemoresistance and is associated with unfavorable survival outcomes in COAD patients.

Univariate analysis by the Cox regression model found that several clinicopathological parameters were associated with survival outcome in COAD patients, including pT stage, pN stage, LVI, PNI, postoperative chemotherapy and tumor EGFR. Furthermore, the immune checkpoint proteins PD-L1, PD-L2 and B7-H3 were also associated with patient survival outcomes. Patients with low expression of tumor PD-L1 and PD-L2 had a higher risk for poor DFS: low tumor PD-L1 (HR=1.656, 95% CI=1.202-2.280, *p*=0.02) and low tumor PD-L2 (HR=1.296, 95% CI=0.955-1.759, *p*=0.096). In contrast, patients with high tumor B7-H3 had an increased risk for a low 5-year DFS (HR=1.657, 95% CI=1.246-2.203, *p*=0.001) compared with that of patients with low tumor B7-H3 expression (Table 1). These results showed that immune checkpoint proteins have significant prognostic relevance for colon adenocarcinoma patients. After adjustment for clinicopathologic parameters, multivariate analysis showed that tumor B7-H3 can be considered an independent prognostic factor for colon adenocarcinoma patients (HR=1.492, 95% CI= 1.114-1.997, *p*=0.007, Table 1). Furthermore, we found that elevated tumor B7-H3 expression was associated with a high risk of poor 5-year DFS in stage II-III patients who received postoperative adjuvant chemotherapy (HR=2.202, 95% CI=1.295-3.744, *p*=0.004, Table S3), suggesting that B7-H3 may limit the therapeutic efficacy of adjuvant chemotherapy.

### B7H3 activated EGFR-ERK signaling to promote chemoresistance

**Figure 3:**
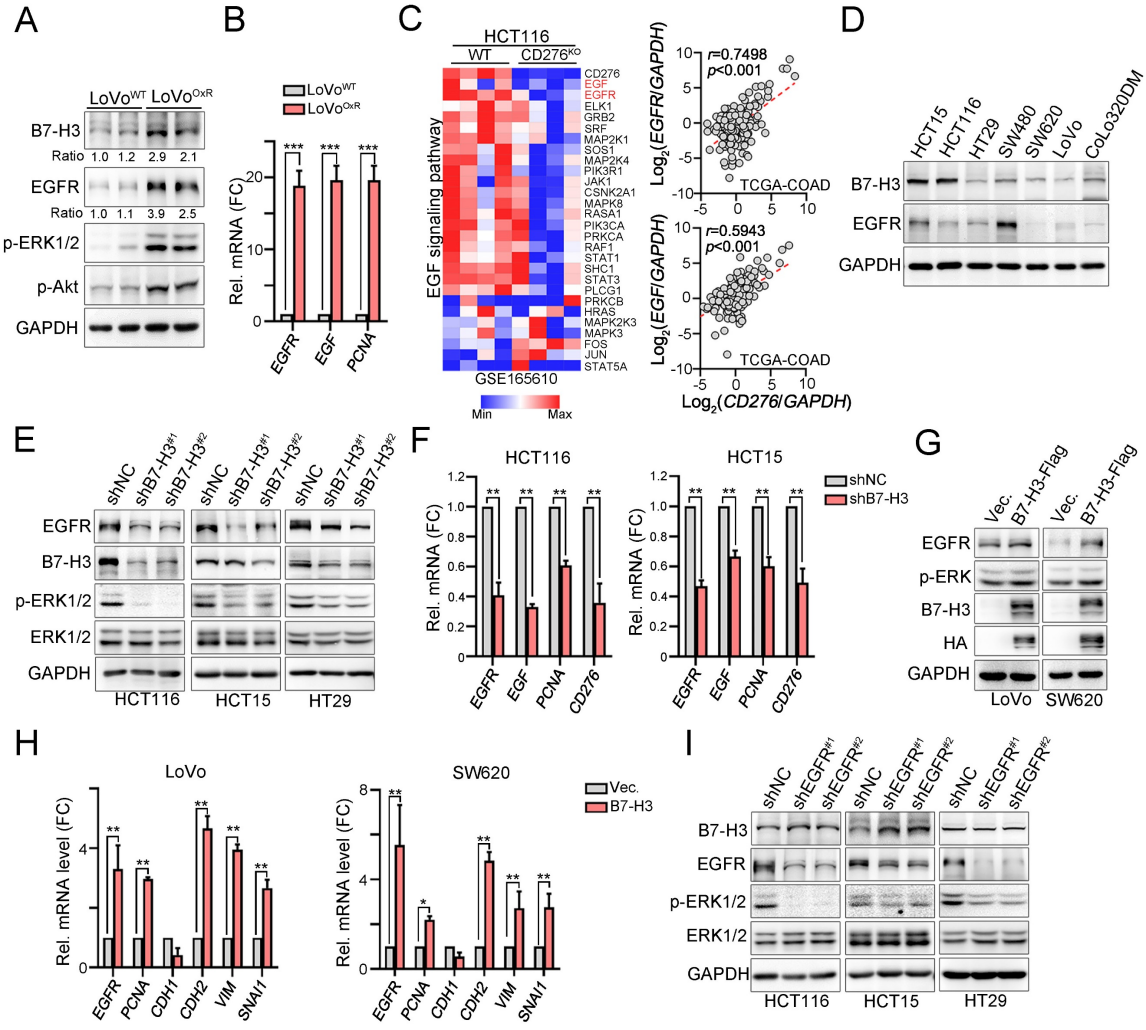
** B7-H3 directly activated EGFR-ERK signaling for cancer cell growth and metastasis.** A. LoVo^WT^ and OXP-resistant cells (LoVo^OxR^) were harvested for immunoblotting (*n*=3). One-Way ANOVA analysis. B. The mRNA expression of *EGF*, *EGFR* and* PCNA* was evaluated by qRT-PCR (*n*=3). One-Way ANOVA analysis. ***p<0.001. C. Heatmap showed that CD276 knockout significantly downregulated EGFR signaling pathway in HCT116 cell line. Data retrieved from GSE165610. *CD276* mRNA level status was positively correlated with the *EGFR* and *EGF* mRNA expression in COAD database in TCGA (*p*<0.001). D. The expression of B7-H3 and EGFR in colorectal cancer cell lines. E. HCT116, HCT15 and HT29 cells were infected with lentivirus carrying shRNA against CD276 and selected by puromycin for three days. The expression of B7-H3, EGFR and p-ERK1/2 was analyzed by immunoblotting (*n*=3). F. HCT116 and HCT15 cells were infected with lentivirus carrying shRNA against CD276 and selected by puromycin for three days. The mRNA expression of B7-H3, EGF and EGFR was analyzed by qRT-PCR (*n*=3). One-Way ANOVA analysis. **p<0.01. G. LoVo were transfected with pCMV-B7-H3-flag for two days. The expression of B7-H3 was analyzed by immunoblotting. The mRNA expression of EGFR and EMT markers was analyzed by qRT-PCR (*n*=3). H. SW620 were transfected with pCMV-B7-H3-flag for two days. The expression of B7-H3 was analyzed by immunoblotting. The mRNA expression of EGFR and EMT markers was analyzed by qRT-PCR (*n*=3). One-Way ANOVA analysis. *p<0.05 and **p<0.01. I. HCT116, HCT15 and HT29 cells were infected with lentivirus carrying shRNA against EGFR and selected by puromycin for three days. The expression of B7-H3, EGFR and p-ERK1/2 was analyzed by immunoblotting.

Because the clinical results showed that tumor B7-H3 expression was positively correlated with tumor EGFR expression and might be associated with the therapeutic efficacy of adjuvant chemotherapy, we first evaluated the level of B7-H3 in LoVo and oxaliplatin (OXP)-resistant LoVo resected tumor tissues that were previously established 35. Immunoblotting results from these resected tumor tissues revealed high B7-H3 and EGFR expression in the LoVo^OXR^ group (Fig. 3A). The mRNA levels of *EGFR*, *EGF* and *PCNA* were also increased (Fig. 3B). Previous studies have indicated that B7-H3-induced signaling and EGFR signaling at least partly share common downstream signaling cascades 38, 39. We therefore put extensive effort into identifying whether the potential mechanisms underlying B7-H3-mediated chemoresistance are mediated through the EGFR/ERK axis. We first compared and analyzed the results of RNA-seq on HCT116^WT^ and HCT116^CD276KO^ cells, which were retrieved from GSE165610 40. We found that EGF signaling was significantly downregulated in HCT116^CD276KO^ cells compared to that in HCT116^WT^ cells (Fig. 3E).

Moreover, this positive correlation between B7-H3 and EGFR/EGF was also observed in the TCGA-COAD database (Fig. 3C, *n*=592). Similarly, we found a positive correlation between B7-H3 and EGFR in colorectal cancer cell lines (Fig. 3D). We then aimed to identify the relationship between B7-H3 and EGFR. As shown in Fig. 3E, we found that knockdown of B7-H3 significantly decreased the level of EGFR and ERK phosphorylation in three CRC cell lines. The mRNA levels of *EGFR*, *EGF* and *PCNA* were also decreased in HCT116 and HCT15 cell lines (Fig. 3F).

In contrast, overexpression of B7-H3 markedly increased the expression of EGFR (Fig. 3G) and the mRNA levels of *EGFR* and *PCNA* in LoVo and SW620 cell lines (Fig. 3H). Furthermore, we found increased expression of EMT markers, including N-cadherin (*CDH2*), vimentin (*VIM*) and Snail (*SNAL1*, Fig. 3H), suggesting that B7-H3 may enhance the EGFR-ERK signaling pathway for cancer cell growth and metastasis. However, we found no change on the protein level of B7-H3 in EGFR-silenced cell lines (Fig. 3I), suggesting that B7-H3 may stabilize EGFR signaling for downstream signaling cascade activation to enhance cancer cell growth and metastasis.

**Figure 4:**
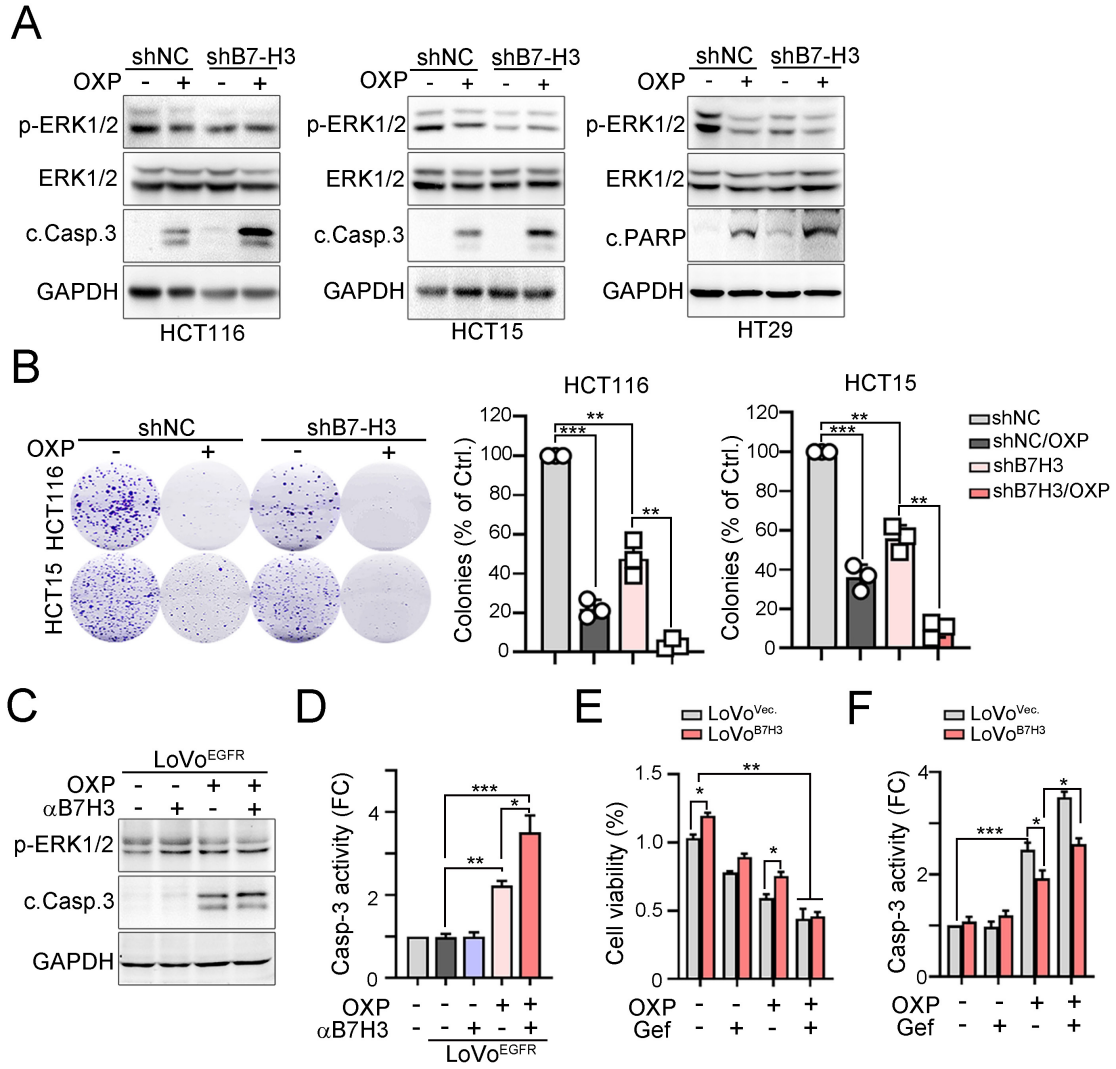
** B7-H3 directly activated EGFR-ERK signaling to promote chemoresistance.** A. Knockdown of B7-H3 significantly decreased the level of phosphor-ERK and increased the cleavage of caspase-3 in response to OXP treatment (30μM, *n*=3). B. Knockdown of B7-H3 significantly decreased the cell viability in response to OXP treatment (30 μM, *n*=3). One-Way ANOVA analysis. **p<0.01 and ***p<0.001. C. LoVo cells were transfected with pCMV-EGFR-flag for 24 hr and treated with OXP (30 μM) and anti-B7-H3 antibodies for 24 hr. The expression of p-ERK1/2 and cleaved casp.3 was analyzed by immunoblotting. D. LoVo cells were transfected with pCMV-EGFR-flag for 24 hour and treated with OXP (30 μM) and anti-B7-H3 antibodies for 24 hr. The activity of Casp.3 was analyzed by caspase-3 activity kit (*n*=3). *p<0.05, **p<0.01 and ***p<0.001. E. LoVo cells were transfected with pCMV-B7-H3-flag for 24 hr and treated with OXP (30 μM) and gefitinib (30 μM) for 24 hr. Cell viability was determined by CCK8 assay (*n*=3). *p<0.05 and **p<0.01. F. LoVo cells were transfected with pCMV-B7-H3-flag for 24 hr and treated with OXP (30 μM) and gefitinib (30 μM) for 24 hr. Cell death was determined by caspase-3 activity assay (*n*=3). *p<0.05 and ***p<0.001.

To further confirm that the expression of B7-H3 is associated with the response to chemotherapeutic drugs, we treated B7-H3-silenced cells with OXP to evaluate its effects (Fig. 4A). Immunoblotting results showed that phosphorylation of ERK was significantly decreased and cleavage of caspase-3 was significantly increased in these three B7-H3-silenced cell lines exposed to OXP (Fig. 4A). The colony formation assay also showed that fewer colonies survived after chemotherapy treatment in B7-H3-silenced cells (Fig. 4B). These results indicated that tumor B7-H3 expression is associated with the response to OXP. Similarly, by neutralizing tumor B7-H3 with anti-B7-H3 antibodies, we found that cancer cells significantly increased susceptibility to OXP, leading to caspase-3 cleavage and activation in EGFR-overexpressing cells (Fig. 4C and 4D). Consistently, targeting EGFR signaling with the small molecule gefitinib also enhanced the response to OXP in B7-H3-overexpressing cells, leading to a decrease in cell viability and caspase-3 activation (Fig. 4E and 4F). Overall, these results indicated that inhibition of EGFR markedly decreased B7-H3-induced chemoresistance in CRC.

### Inhibition of EGFR signaling markedly increased the response to OXP in B7-H3-overexpressing CRC* in vivo*

**Figure 5:**
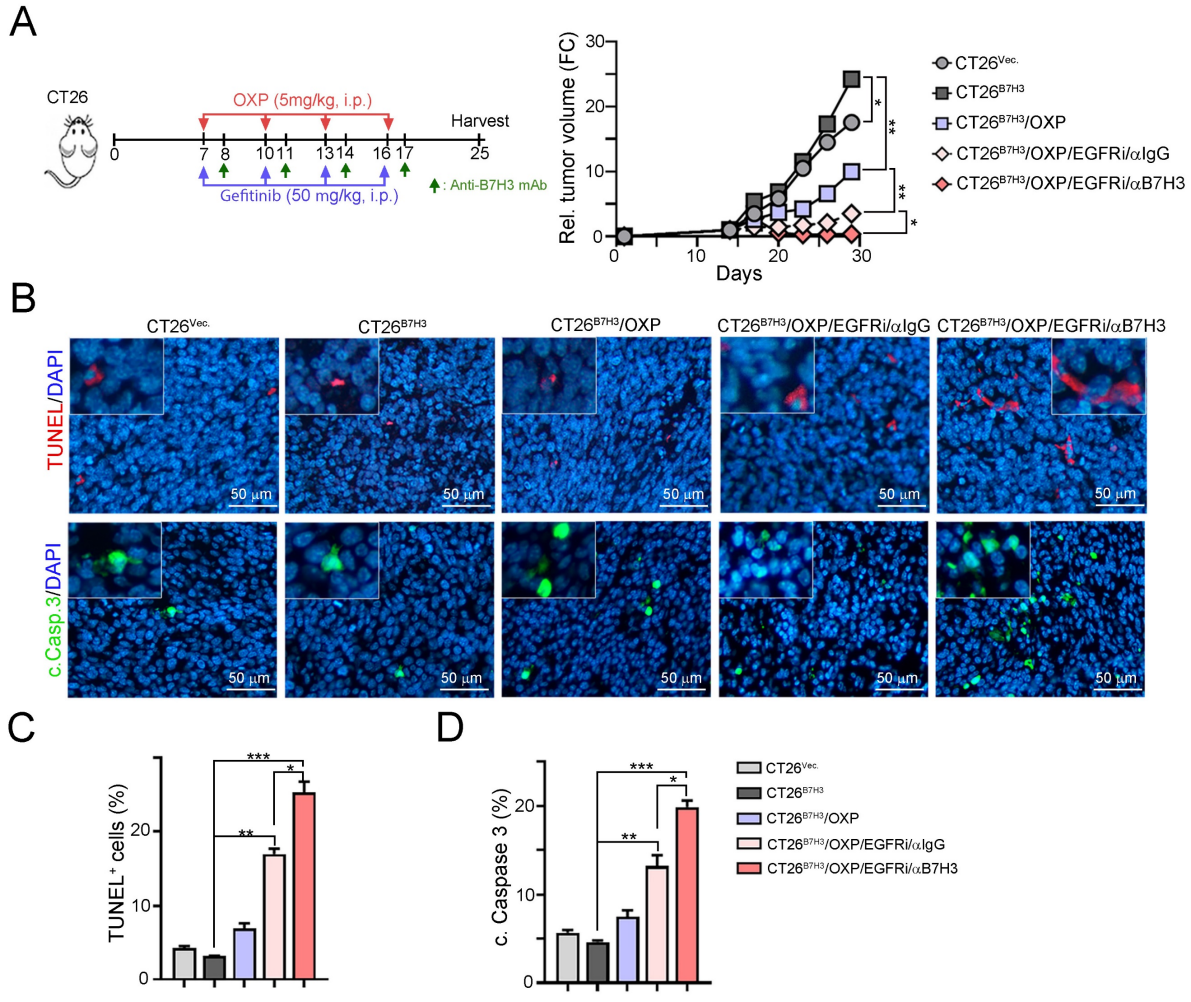
** Inhibition of EGFR significantly decreased B7-H3-induced chemoresistance to OXP *in vivo*.** A. The schema of gefitinib and OXP treatment. CT26-Vec. and CT26-B7-H3 cells were subcutaneous injected into BALB/c nude mice for 7 days. OXP (5 mg/kg), gefitinib (50 mg/kg) and anti-B7-H3 antibodies (100 μg/ mouse) were given on indicated days (*n*=5/group). Tumor volume was recorded every three days. Two-Way ANOVA analysis. *p<0.05 and **p<0.01. B. The resected tumor tissues were analyzed by IHC to examine the percentage of apoptotic cells (TUNEL^+^ cells and cleaved caspase-3). One-Way ANOVA analysis. *p<0.05. C. The percentage of apoptotic cells (TUNEL^+^ cells) was quantified (*n*=3). One-Way ANOVA analysis. *p<0.05, **p<0.01 and ***p<0.001. D. The percentage of cleaved caspase-3-positive cells was quantified (*n*=3). One-Way ANOVA analysis. *p<0.05, **p<0.01 and ***p<0.001.

To further demonstrate that EGFR/ERK signaling influences B7-H3-induced chemoresistance, we generated a B7-H3-overexpressing CT26 cell line and inoculated it into BALB/c nude mice. OXP, gefitinib and anti-B7-H3 antibodies were given on the indicated days (Fig. 5A). As shown in Fig. 5A, tumor growth quickly increased in CT26^B7H3^ tumor-bearing mice. The tumor volume was reduced in response to OXP in CT26^B7H3^-bearing mice. However, the tumor volume was significantly decreased by OXP in combination with the EGFR inhibitor gefitinib. The tumor volume was markedly decreased by triple treatment with OXP, gefitinib and anti-B7-H3 (Fig. 5A and 5B). Furthermore, the TUNEL^+^ apoptotic cells and cleaved caspase-3^+^ apoptotic cells were significantly increased in the triple treatment group (Fig. 5C and 5D). Overall, these results demonstrated that dual targeting of B7H3 and EGFR significantly enhanced the susceptibility to OXP and delayed tumor growth in CRC.

## Discussion

In the present study, we found that B7-H3 showed higher expression in COAD patients than in tumor PD-L1 and PD-L2 patients. High B7-H3 expression was associated with the risk of distant metastasis and poor survival outcomes. Moreover, we found high tumor B7-H3 expression in the low PD-L1 subgroup, suggesting that B7-H3 is a suitable immunotherapeutic target in COAD patients. Additionally, we found that recurrent tumors exhibited higher B7-H3 expression than primary tumors after treatment with postoperative adjuvant chemotherapy, suggesting that tumor B7-H3 was associated with resistance to adjuvant chemotherapy and acted as an independent prognostic factor, especially in high-risk COAD patients. Moreover, we found that B7-H3 induced chemoresistance via EGFR/ERK signaling. Inhibition of EGFR by gefitinib significantly rescued the response to OXP in B7-H3-overexpressing CRC cells *in vitro* and *in vivo*. These results indicated that dual targeting of B7-H3 and EGFR may be a valid strategy in combination with anti-B7-H3 antibodies for COAD patients.

Several studies have found that B7-H3 mainly acts as an inhibitory protein to create an immunosuppressive microenvironment with other immune checkpoints 19. We found that B7-H3 expression (49.9%) was more abundant than PD-L1 (33.6%) and PD-L2 (34%) expression. Moreover, we found that high expression of tumor B7-H3 was significantly increased in patients with low tumor PD-L1, which was not suitable for anti-PD1/PD-L1 antibodies. Indeed, previous studies showed that the coexpression of B7-H3 and PD-L1 was relatively low in NSCLC and SCLC 11, 12. Altan et al. showed that the frequency of coexpressed B7-H3 and PD-L1 was relatively low, with 17.6% of NSCLC 11. Similar to the finding in NSCLC, limited correlation with PD-L1, B7-H3 and B7-H4 in SCLC also showed minimal coexpression. Lu et al. also showed that 29.2% of CRC patients expressed PD-L1 and 50.9% of CRC patients exhibited B7-H3, which supports our findings 13. They found that 5.7% of CRC patients coexpressed B7-H3 and PD-L1. Therefore, the mutually exclusive expression pattern may show mechanistic differences that affect the tumor microenvironment and tumor immune evasion, leading to a reduced therapeutic response to anti-PD1/PD-L1 ICIs. These results suggested that CRC may use only one immunosuppressive pathway for tumor escape. Therefore, it may be necessary to stratify CRC patients who are unresponsive or resistant to immune checkpoint inhibitors and provide alternative immunotherapeutic agents.

We found that high expression of tumor B7-H3 increased the risk of tumor relapse in high-risk stage II-III COAD patients. Moreover, we found that residual tumors and metastatic tumors after adjuvant chemotherapy exhibited a higher expression pattern than the primary tumors, suggesting that the standard chemotherapeutic regimen triggered an increase in tumor B7-H3. In addition to its immunomodulatory function, B7-H3 also participates in chemoresistance and metastasis in several malignancies 22, 41-43. Overexpression of B7-H3 affects the therapeutic efficacy of DNA damaging agents such as idarubicin, cytarabine and oxaliplatin 43. B7-H3 also participates in HK2-mediated aerobic glycolysis for chemoresistance. These results revealed that B7-H3-mediated aerobic glycolysis may also be involved in the recurrence of tumors after chemotherapy treatment, suggesting that B7-H3 might be a potential target for preventing the development of CRC chemoresistance.

Similarly, several studies found that PD-L1 was more highly expressed in metastatic tumors than in primary tumors, including in CRC patients 44. Our previous studies found that immunogenic chemotherapy and radiotherapy can increase tumor PD-L1 expression in CRC 45-47. Overexpression of PD-L1 significantly increased resistance to OXP in CRC 48, suggesting that these immunomodulatory proteins also participate in resistance to chemotherapy. Therefore, targeting the B7-H3 signaling pathway may increase the clinical benefit of metastatic CRC patients. Here, we found that B7-H3 and EGFR may share similar signaling pathways to promote chemoresistance. B7-H3 may stabilize EGFR to activate its downstream signaling for the EMT process and chemoresistance. Therefore, targeting EGFR with the small molecule gefitinib markedly increased the response to chemotherapy in B7-H3-overexpressing CRC *in vitro* and *in vivo*. A previous study reported that B7-H3-induced signaling shares a similar signaling pathway with EGFR, which supports our results 49. Ding et al indicated that the B7-H3-induced signaling pathway activated the divergent EGFR signaling pathway, suggesting the translational potential of combined targeted therapy of B7-H3 and EGFR in non-small lung adenocarcinoma 39. Therefore, dual targeting of EGFR and B7-H3 may benefit the therapeutic efficacy of chemotherapy in metastatic CRC patients.

In conclusion, compared with that of the immune checkpoint proteins PD-L1, PD-L2 and B7-H3 exhibited high expression in COAD patients, especially in patients with lower PD-L1. Furthermore, B7-H3 expression in primary tumors was significantly associated with a high risk of distant metastasis and poor survival. Targeting EGFR signaling may partially rescue the response to chemotherapy in B7-H3-overexpressing COAD patients, providing potential therapeutic strategies for dual targeting of B7-H3 and EGFR to increase the clinical benefit in COAD patients.

## Supplementary Material

Supplementary figure and tables.

## Figures and Tables

**Table 1:** Univariate and multivariate analysis of DFS and known prognostic factors in colon carcinoma patients.

Parameters	Univariate analysis						Multivariate analysis		
	No. at risk^a^	Events	HR	95% CI	*p* value		HR	95% CI	*p* value
Sex	429	197							
Female	204	95	1.0						
Male	225	102	0.957	0.724-1.266	0.758				
Age									
<65	208	91	1						
≥65	221	106	1.17	0.884-1.548	0.272				
pT stage									
pT1-2	71	17	1				1		
pT3-4	358	180	2.566	1.560-4.221	**<0.001**		1.599	0.932-2.744	**0.088**
pN stage									
Negative	223	71	1				1		
Positive	206	126	2.475	1.848-3.315	**<0.001**		2.404	1.645-3.513	<0.001
Tumor location									
Distal colon	181	83	1						
Proximal colon	243	109	0.95	0.714-1.264	0.727				
Tumor differentiation									
Well to moderate	419	191	1						
Poor	6	3	0.989	0.316-3.095	0.985				
Lymphovascular invasion									
Absent	184	58	1				1		
Present	245	139	2.189	1.611-2.976	**<0.001**		1.386	0.970-1.982	**0.073**
Perineural invasion									
Absent	260	90	1				1		
Present	168	106	2.244	1.693-2.975	**<0.001**		1.616	1.179-2.215	**0.003**
Post-operative chemotherapy									
No	175	64	1				1		
Yes	254	133	1.524	1.131-2.054	**0.006**		0.578	0.392-0.852	**0.006**
Tumor EGFR									
Negative	126	76	1						
Positive	18	16	2.106	1.224-3.623	**0.007**				
KRAS									
WT	98	70	1						
Mutation	56	41	1.065	0.724-1.566	0.750				
Tumor B7H3									
Low	215	80	1				1		
High	214	117	1.657	1.246-2.203	**0.001**		1.492	1.114-1.997	**0.007**
Tumor PD-L1									
High	144	51	1				1		
Low	284	146	1.656	1.204-2.280	**0.020**		1.674	1.212-2.311	**0.002**
Tumor PD-L2									
High	146	60	1						
Low	274	132	1.296	0.955-1.759	0.096				

^a^: Number of cases may differ due to missing data.

## Abbreviations

5-FU: 5-fluorouracil
B7-H3: B7 homology 3
BRCC3: BRCA1/BRCA2-containing complex subunit 3
CDH2: N-cadherin
COAD: colon adenocarcinoma
CRC: colorectal cancer
DFS: disease-free survival
EMT: epithelial-to-mesenchymal transition
OS: overall survival
OXP: oxaliplatin
VIM: vimentin
XRCC1: X-ray repair cross-complementing Group 1

## References

[1] Sung H, Ferlay J, Siegel RL, Laversanne M, Soerjomataram I, Jemal A (2021). Global Cancer Statistics 2020: GLOBOCAN Estimates of Incidence and Mortality Worldwide for 36 Cancers in 185 Countries. CA Cancer J Clin.

[2] Siegel RL, Miller KD, Goding Sauer A, Fedewa SA, Butterly LF, Anderson JC (2020). Colorectal cancer statistics, 2020. CA Cancer J Clin.

[3] Van Cutsem E, Cervantes A, Nordlinger B, Arnold D, Group EGW (2014). Metastatic colorectal cancer: ESMO Clinical Practice Guidelines for diagnosis, treatment and follow-up. Ann Oncol.

[4] Benitez Majano S, Di Girolamo C, Rachet B, Maringe C, Guren MG, Glimelius B (2019). Surgical treatment and survival from colorectal cancer in Denmark, England, Norway, and Sweden: a population-based study. Lancet Oncol.

[5] Zhou WT, Jin WL (2021). B7-H3/CD276: An Emerging Cancer Immunotherapy. Front Immunol.

[6] Lee YH, Martin-Orozco N, Zheng P, Li J, Zhang P, Tan H (2017). Inhibition of the B7-H3 immune checkpoint limits tumor growth by enhancing cytotoxic lymphocyte function. Cell Res.

[7] Yonesaka K, Haratani K, Takamura S, Sakai H, Kato R, Takegawa N (2018). B7-H3 Negatively Modulates CTL-Mediated Cancer Immunity. Clin Cancer Res.

[8] Inamura K, Takazawa Y, Inoue Y, Yokouchi Y, Kobayashi M, Saiura A (2018). Tumor B7-H3 (CD276) Expression and Survival in Pancreatic Cancer. J Clin Med.

[9] Crispen PL, Sheinin Y, Roth TJ, Lohse CM, Kuntz SM, Frigola X (2008). Tumor cell and tumor vasculature expression of B7-H3 predict survival in clear cell renal cell carcinoma. Clin Cancer Res.

[10] Sun J, Chen LJ, Zhang GB, Jiang JT, Zhu M, Tan Y (2010). Clinical significance and regulation of the costimulatory molecule B7-H3 in human colorectal carcinoma. Cancer Immunol Immunother.

[11] Altan M, Pelekanou V, Schalper KA, Toki M, Gaule P, Syrigos K (2017). B7-H3 Expression in NSCLC and Its Association with B7-H4, PD-L1 and Tumor-Infiltrating Lymphocytes. Clin Cancer Res.

[12] Carvajal-Hausdorf D, Altan M, Velcheti V, Gettinger SN, Herbst RS, Rimm DL (2019). Expression and clinical significance of PD-L1, B7-H3, B7-H4 and TILs in human small cell lung Cancer (SCLC). J Immunother Cancer.

[13] Lu Z, Zhao ZX, Cheng P, Huang F, Guan X, Zhang MG (2020). B7-H3 immune checkpoint expression is a poor prognostic factor in colorectal carcinoma. Mod Pathol.

[14] Seaman S, Zhu Z, Saha S, Zhang XM, Yang MY, Hilton MB (2017). Eradication of Tumors through Simultaneous Ablation of CD276/B7-H3-Positive Tumor Cells and Tumor Vasculature. Cancer Cell.

[15] Zhang C, Zhang Z, Li F, Shen Z, Qiao Y, Li L (2018). Large-scale analysis reveals the specific clinical and immune features of B7-H3 in glioma. Oncoimmunology.

[16] Aung PP, Parra ER, Barua S, Sui D, Ning J, Mino B (2019). B7-H3 Expression in Merkel Cell Carcinoma-Associated Endothelial Cells Correlates with Locally Aggressive Primary Tumor Features and Increased Vascular Density. Clin Cancer Res.

[17] Bin Z, Guangbo Z, Yan G, Huan Z, Desheng L, Xueguang Z (2014). Overexpression of B7-H3 in CD133+ colorectal cancer cells is associated with cancer progression and survival in human patients. J Surg Res.

[18] Zhang T, Wang F, Wu JY, Qiu ZC, Wang Y, Liu F (2018). Clinical correlation of B7-H3 and B3GALT4 with the prognosis of colorectal cancer. World J Gastroenterol.

[19] Dong P, Xiong Y, Yue J, Hanley SJB, Watari H (2018). B7H3 As a Promoter of Metastasis and Promising Therapeutic Target. Front Oncol.

[20] Jiang B, Zhang T, Liu F, Sun Z, Shi H, Hua D (2016). The co-stimulatory molecule B7-H3 promotes the epithelial-mesenchymal transition in colorectal cancer. Oncotarget.

[21] Flem-Karlsen K, Tekle C, Andersson Y, Flatmark K, Fodstad O, Nunes-Xavier CE (2017). Immunoregulatory protein B7-H3 promotes growth and decreases sensitivity to therapy in metastatic melanoma cells. Pigment Cell Melanoma Res.

[22] Zhang P, Chen Z, Ning K, Jin J, Han X (2017). Inhibition of B7-H3 reverses oxaliplatin resistance in human colorectal cancer cells. Biochem Biophys Res Commun.

[23] Liu Z, Zhang W, Phillips JB, Arora R, McClellan S, Li J (2019). Immunoregulatory protein B7-H3 regulates cancer stem cell enrichment and drug resistance through MVP-mediated MEK activation. Oncogene.

[24] Zhang W, Wang Y, Wang J, Dong F, Zhu M, Wan W (2015). B7-H3 silencing inhibits tumor progression of mantle cell lymphoma and enhances chemosensitivity. Int J Oncol.

[25] Sun ZZ, Zhang T, Ning K, Zhu R, Liu F, Tang SC (2016). B7-H3 upregulates BRCC3 expression, antagonizing DNA damage caused by 5-Fu. Oncol Rep.

[26] Du H, Hirabayashi K, Ahn S, Kren NP, Montgomery SA, Wang X (2019). Antitumor Responses in the Absence of Toxicity in Solid Tumors by Targeting B7-H3 via Chimeric Antigen Receptor T Cells. Cancer Cell.

[27] Majzner RG, Theruvath JL, Nellan A, Heitzeneder S, Cui Y, Mount CW (2019). CAR T Cells Targeting B7-H3, a Pan-Cancer Antigen, Demonstrate Potent Preclinical Activity Against Pediatric Solid Tumors and Brain Tumors. Clin Cancer Res.

[28] Huang KC, Chiang SF, Chen TW, Chen WT, Yang PC, Ke TW (2020). Prognostic relevance of programmed cell death 1 ligand 2 (PDCD1LG2/PD-L2) in patients with advanced stage colon carcinoma treated with chemotherapy. Sci Rep.

[29] Huang CY, Chiang SF, Ke TW, Chen TW, You YS, Chen WT (2018). Clinical significance of programmed death 1 ligand-1 (CD274/PD-L1) and intra-tumoral CD8+ T-cell infiltration in stage II-III colorectal cancer. Sci Rep.

[30] Lin TY, Fan CW, Maa MC, Leu TH (2015). Lipopolysaccharide-promoted proliferation of Caco-2 cells is mediated by c-Src induction and ERK activation. Biomedicine (Taipei).

[31] Wang X, Sheu JJ, Lai MT, Yin-Yi Chang C, Sheng X, Wei L (2018). RSF-1 overexpression determines cancer progression and drug resistance in cervical cancer. Biomedicine (Taipei).

[32] Uhlen M, Fagerberg L, Hallstrom BM, Lindskog C, Oksvold P, Mardinoglu A (2015). Proteomics. Tissue-based map of the human proteome. Science.

[33] Uhlen M, Zhang C, Lee S, Sjostedt E, Fagerberg L, Bidkhori G (2017). A pathology atlas of the human cancer transcriptome. Science.

[34] Hoadley KA, Yau C, Hinoue T, Wolf DM, Lazar AJ, Drill E (2018). Cell-of-Origin Patterns Dominate the Molecular Classification of 10,000 Tumors from 33 Types of Cancer. Cell.

[35] Huang CY, Chiang SF, Chen WT, Ke TW, Chen TW, You YS (2018). HMGB1 promotes ERK-mediated mitochondrial Drp1 phosphorylation for chemoresistance through RAGE in colorectal cancer. Cell Death Dis.

[36] Huang KC, Chiang SF, Chang HY, Chen WT, Yang PC, Chen TW (2022). Engineered sTRAIL-armed MSCs overcome STING deficiency to enhance the therapeutic efficacy of radiotherapy for immune checkpoint blockade. Cell Death Dis.

[37] Huang KC, Lai CY, Hung WZ, Chang HY, Lin PC, Chiang SF (2023). A Novel Engineered AAV-Based Neoantigen Vaccine in Combination with Radiotherapy Eradicates Tumors. Cancer Immunol Res.

[38] Wang S, Zhang X, Ning H, Dong S, Wang G, Sun R (2022). B7 homolog 3 induces lung metastasis of breast cancer through Raf/MEK/ERK axis. Breast Cancer Res Treat.

[39] Ding M, Liao H, Zhou N, Yang Y, Guan S, Chen L (2020). B7-H3-Induced Signaling in Lung Adenocarcinoma Cell Lines with Divergent Epidermal Growth Factor Receptor Mutation Patterns. Biomed Res Int.

[40] Durlanik S, Fundel-Clemens K, Viollet C, Huber HJ, Lenter M, Kitt K (2021). CD276 is an important player in macrophage recruitment into the tumor and an upstream regulator for PAI-1. Sci Rep.

[41] Zhang B, Cheng B, Li FS, Ding JH, Feng YY, Zhuo GZ (2015). High expression of CD39/ENTPD1 in malignant epithelial cells of human rectal adenocarcinoma. Tumour Biol.

[42] Shi T, Ma Y, Cao L, Zhan S, Xu Y, Fu F (2019). B7-H3 promotes aerobic glycolysis and chemoresistance in colorectal cancer cells by regulating HK2. Cell Death Dis.

[43] Zhang W, Wang J, Wang Y, Dong F, Zhu M, Wan W (2015). B7-H3 silencing by RNAi inhibits tumor progression and enhances chemosensitivity in U937 cells. Onco Targets Ther.

[44] Garcia-Diez I, Hernandez-Ruiz E, Andrades E, Gimeno J, Ferrandiz-Pulido C, Yebenes M (2018). PD-L1 Expression is Increased in Metastasizing Squamous Cell Carcinomas and Their Metastases. Am J Dermatopathol.

[45] Huang KC, Chiang SF, Chen WT, Chen TW, Hu CH, Yang PC (2020). Decitabine Augments Chemotherapy-Induced PD-L1 Upregulation for PD-L1 Blockade in Colorectal Cancer. Cancers (Basel).

[46] Huang KC-Y, Chiang S-F, Ke T-W, Chen T-W, Hu C-H, Yang P-C (2021). DNMT1 constrains IFNβ-mediated anti-tumor immunity and PD-L1 expression to reduce the efficacy of radiotherapy and immunotherapy. OncoImmunology.

[47] Huang KC, Chiang SF, Yang PC, Ke TW, Chen TW, Hu CH (2021). Immunogenic Cell Death by the Novel Topoisomerase I Inhibitor TLC388 Enhances the Therapeutic Efficacy of Radiotherapy. Cancers (Basel).

[48] Chen TW, Hung WZ, Chiang SF, Chen WT, Ke TW, Liang JA (2022). Dual inhibition of TGFbeta signaling and CSF1/CSF1R reprograms tumor-infiltrating macrophages and improves response to chemotherapy via suppressing PD-L1. Cancer Lett.

[49] Ren X, Li Y, Nishimura C, Zang X (2022). Crosstalk between the B7/CD28 and EGFR pathways: Mechanisms and therapeutic opportunities. Genes Dis.

