# Supplementary material for: Dual Inhibition of B7-H3 and EGFR Overcomes Acquired Chemoresistance in Colon Adenocarcinoma
Source: J Cancer. 2024 Feb 4;15(6):1750–61. doi: 10.7150/jca.91089 (PMC10869969; doi:10.7150/jca.91089)
Supplement: Supplementary file 1 — Supplementary figure and tables. [file jcav15p1750s1.pdf]

Supplementary information

Dual Inhibition of B7-H3 and EGFR Overcomes Acquired Chemoresistance in Colon Adenocarcinoma

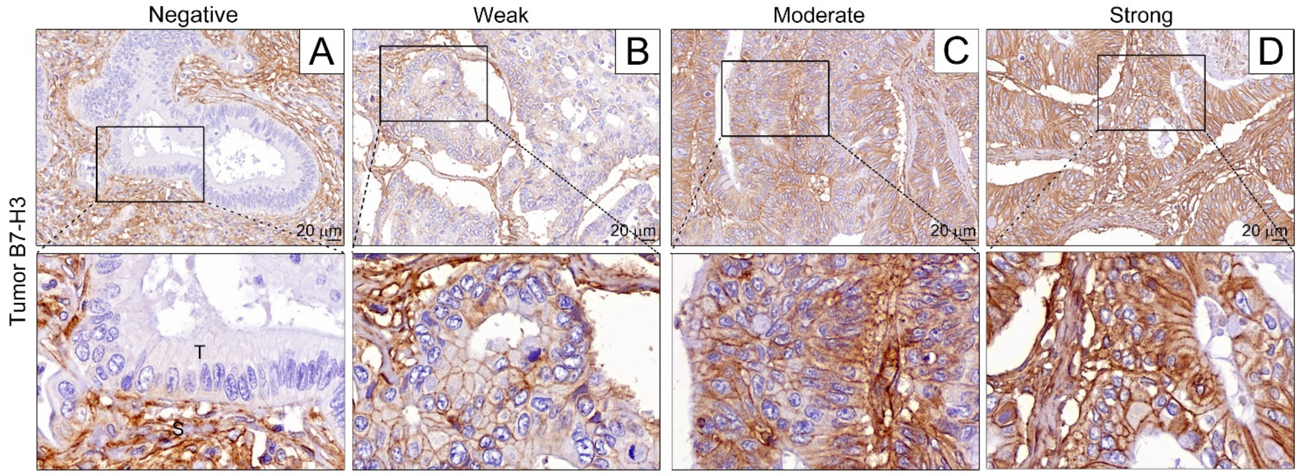

Figure S1. Expression patterns of tumor B7-H3 within the tumor microenvironment of colon adenocarcinoma. (**A**) Negative tumor B7-H3 immunohistochemical staining within the tumor microenvironment. (**B**) Weak membranous B7-H3 expression in tumor cells. (**C**) Moderate membranous B7-H3 expression in tumor cells. (**D**) Strong tumor membranous B7-H3 expression. Scale bar = 20 μm. T: tumor cells, S: stromal cells.

Table S1. Clinicopathological parameters of colon carcinoma patients (n=429)

Clinicopathological parameters	Total no.	Tumor B7H3		*p* value	Stromal B7H3		*p* value	PD-L1		*p* value	PD-L2		*p* value
		Low	High		Low	High		Low	High		Low	High	
	429	215	214		203	226		284	144		274	146	
Gender				0.811			0.624			0.589			0.866
Female	204	101	103		94	110		138	66		129	70	
Male	225	114	111		109	116		146	78		145	76	
Age				0.531			0.376			0.736			0.09
<65	208	101	107		103	105		139	68		142	63	
≥65	221	114	107		100	121		145	76		132	83	
Tumor location				0.9			0.728			0.011			0.095
Proximal colon	181	92	89		88	93		108	73		124	53	
Distal colon	243	122	121		114	129		173	69		148	90	
Unspecified	5	1	4		1	4		3	2		2	3	
pT stage				0.008			0.001			0.245			0.429
pT1	30	20	10		22	8		23	6		19	11	
pT2	41	28	13		24	17		23	18		25	15	
pT3	266	129	137		105	161		176	90		166	96	
pT4	92	38	54		52	40		62	30		64	24	
pN stage				0.027			0.004			0.832			0.001
N0	223	119	104		92	131		150	72		141	78	
N1	112	44	68		53	59		72	40		60	49	
N2	94	52	42		58	36		62	32		73	19	
TNM stage (7th AJCC)				0.019			0.001			0.188			0.224
Stage I	57	39	18		40	17		40	16		37	19	
Stage II	159	80	79		48	111		104	55		98	58	
Stage III	126	58	68		56	70		76	50		75	47	
Stage IV	87	38	49		59	28		64	23		64	22	
Tumor differentiation				0.41			0.80			0.39			0.54
Well	22	8	14		15	7		17	5		13	9	
Moderate	396	202	194		182	214		261	134		253	135	
Poor	6	3	3		4	2		3	3		5	1	
Unknown	5	2	3		2	3		3	2		3	1	
Lymphovascular invasion				0.035			0.549			0.745			0.231
Absent	184	103	81		84	100		123	60		111	68	
Present	245	112	133		119	126		161	84		163	78	
Perineural invasion				0.1658			0.591			0.363			0.087
Absent	260	137	123		120	140		176	83		173	80	
Present	168	77	91		82	86		107	61		100	66	
Unknown	1	1	0		1	0		1	0		1	0	
Tumor relapse				<0.0001			0.539			0.066			0.291
Absent	352	199	153		169	183		226	125		228	117	
Present	77	16	61		34	43		58	19		46	31	
EGFR				0.019			0.734			0.08			0.075
Negative	126	72	54		86	40		94	31		85	39	
Positive	18	5	13		13	5		10	8		16	2	
KRAS				0.44			0.691			0.375			0.573
WT	98	43	55		54	44		65	33		62	34	
Mutation	56	21	35		29	27		41	15		38	17	
MMR status				0.466			0.455			0.06			0.001
Proficient	395	200	195		189	206		267	128		242	145	
Deficient	34	15	19		14	20		17	16		32	1	
Post-operative chemotherapy				0.001			0.97			0.815			0.483
No	175	104	71		83	92		106	56		103	60	
Yes	254	111	143		120	134		173	87		171	86	

Pearson test was used, and Fisher's exact test was used when counts were less than 5. The test did not include the “unknown or unspecified" group. Only stage I-III patients were included in the statistical analysis on LR, DM and tumor relapse.

Table S2. Correlation between clinicopathologic parameters and 5-year OS

Parameters	No[a]	5-year DFS (%)	*p* value*	5-year OS %	*p* value*
	429	54.0%		60.4%	
Sex			0.758		0.651
Female	204	53.4%		61.3%	
Male	225	54.5%		59.6%	
Age			0.271		0.097
<65	208	56.3%		63.9%	
≥65	221	51.8%		57.0%	
pT stage			<0.001		<0.001
pT1-2	71	76.1%		78.9%	
pT3-4	358	49.6%		56.7%	
pN stage			<0.001		<0.001
Negative	223	68.0%		72.6%	
Positive	206	38.8%		47.1%	
Tumor location			0.727		0.267
Proximal colon	181	54.1%		58.6%	
Distal colon	243	55.0%		62.6%	
Tumor differentiation			0.985		0.703
Well to moderate	419	54.3%		60.6%	
Poor	6	50.0%		50.0%	
Lymphovascular invasion			<0.001		0.001
Absent	184	68.3%		72.8%	
Present	245	43.3%		51.0%	
Perineural invasion			<0.001		0.001
Absent	260	65.3%		70.0%	
Present	168	36.9%		45.8%	
Post-operative chemotherapy			0.005		0.068
No	175	63.4%		66.9%	
Yes	254	47.4%		55.9%	
Tumor EGFR			<0.006		0.025
Negative	126	39.2%		42.9%	
Positive	18	11.1%		16.7%	
KRAS			0.75		0.21
WT	98	28.6%		43.9%	
Mutation	56	25.5%		33.9%	
Tumor B7H3			<0.001		0.02
Low	215	62.8%		66.5%	
High	214	45.1%		54.2%	
Tumor PD-L1			0.002		0.001
High	144	64.6%		71.5%	
Low	284	48.4%		54.6%	
Tumor PD-L2			0.094		0.003
High	146	58.9%		69.9%	
Low	274	51.6%		55.8%	

[a]Number of cases may differ due to missing data.

Table S3. Multivariate analysis of DFS in high risk stage II-III colon carcinoma patients (n=285).

Parameters		5-year DFS			
	No. at risk[a]	Events	HR	95% CI	*p* value
**Stage II-III (n=285)**					
pT stage					
pT1-2	13	3	1		0.356
pT3-4	272	102	1.745	0.535-5.694	
pN stage					0.248
Negative	159	53	1		
Positive	126	52	1.281	0.841-1.950	
Lymphovascular invasion					0.598
Absent	121	37	1		
Present	164	68	1.128	0.721-1.764	
Perineural invasion					0.051
Absent	180	55	1		
Present	104	49	1.5	0.998-2.255	
Tumor B7H3					0.009
Low	138	39	1		
High	147	66	1.713	1.146-2.561	
**Post-operative chemotherapy (n=181)**					
pT stage					
pT1-2	11	2	1		
pT3-4	170	67	2.134	0.506-9.007	0.302
pN stage					
Negative	69	24	1		
Positive	112	45	1.229	0.735-2.053	0.432
Lymphovascular invasion					
Absent	52	15	1		
Present	129	54	1.317	0.725-2.391	0.365
Perineural invasion					
Absent	99	29	1		
Present	82	40	1.615	0.986-2.643	0.057
Tumor B7H3					
Low	78	19	1		
High	103	50	2.202	1.295-3.744	0.004
**W/o chemotherapy (n=104)**					
pT stage					
pT1-2	2	1	1		
pT3-4	102	35	0.669	0.075-6.009	0.720
pN stage					
Negative	90	29	1		
Positive	14	7	2.066	0.788-5.414	0.140
Lymphovascular invasion					
Absent	69	22	1		
Present	35	14	1.180	0.555-2.508	0.666
Perineural invasion					
Absent	81	26	1		
Present	22	9	1.167	0.500-0.2725	0.721
Tumor B7H3					
Low	60	20	1		
High	44	16	1.257	0.628-2.517	0.519
